# Components of navigation ability and their predictors in a community-dwelling sample of older adults

**DOI:** 10.3389/fragi.2023.1239094

**Published:** 2023-10-19

**Authors:** Michael J. Prevratil, Dorota Kossowska-Kuhn, Nicholas Gray, Neil Charness

**Affiliations:** Department of Psychology, Florida State University, Tallahassee, FL, United States

**Keywords:** navigation, memory, aging, EFA (exploratory factor analysis), wayfinding, cognition

## Abstract

**Introduction:** Navigation, as a complex skill important for independent living, requires a variety of cognitive processes. Current scales tapping components are lengthy and can be burdensome for older adults.

**Methods:** Community-dwelling older adults (*n* = 380, age 60–90 years) completed an online survey tapping wayfinding, being lost navigating, and needing help navigating. Participants then completed objective measures of navigation ability and self-reported memory ability. Cronbach’s *α* was calculated for navigation subscales consisting of subsets of the Wayfinding Questionnaire and Santa Barbara Sense of Direction Questionnaire, and an exploratory factor analysis (EFA) was conducted. Regression analyses were used to test whether objective navigation, memory, and demographic information navigation predicted navigation subscale performance.

**Results:** Each of the individual subscales demonstrated high reliability. EFA generated five unique factors: routing, mental mapping, navigation in near vicinities, feeling lost in far vicinities, and needing help in far vicinities. Across regression analyses, memory, gender, and performance on the Spatial Orientation Test were significant predictors.

**Discussion:** Navigation is a multi-faceted construct that can be reliably measured using concise surveys. Further research is necessary to understand the intricacies of aging and navigation.

## 1 Introduction

The ability to navigate one’s surroundings, to orient oneself in their current environment and move from one location to the next, is a core skill that everyone uses daily. Navigation ability, as a psychological construct, includes spatial orientation, mental rotation, planning, and other components (van der Ham et al., 2020). With normal aging, navigation becomes more difficult. Difficulties present themselves in the form of physical challenges such as reduced motor capacity (VanSwearingen and Studenski, 2014) and mental challenges as a function of age-related declines in spatial abilities, planning, and memory (Harada et al., 2013). Such cognitive difficulties are exacerbated in those older adults with mild cognitive impairment (MCI) and dementia (Plácido et al., 2022). Navigation has also been identified as a marker of progression to MCI (Hort et al., 2007; Costa et al., 2020) and dementia (Lithfous et al., 2013; Coughlan et al., 2018). Spatial navigation is also considered to be a distinct skill individuals possess that is affected separately from other areas of cognition [for further discussion, see Lester et al. (2017) and Moffat (2009)].

When reviewing the literature, two major problems are consistently present. One issue is the diversity of measures for probing navigation abilities. Because of the complexity associated with navigating one’s environment, it is challenging to capture navigation, spatial ability, and spatial orientation within a single measure. For example, two common measures, the “Wayfinding Questionnaire” (de Rooij et al., 2019) and the “Santa Barbara Sense of Direction Questionnaire” (Hegarty et al., 2002) contain approximately twenty items each. Some items in these surveys target aspects of navigation that are less direct (ex., “I have a poor memory for where I left things”). As a result of the need to employ multiple measures, gathering sufficiently comprehensive metrics of navigation becomes time-consuming to conduct both for the experimenter and the participant. Hence, one of our goals is to validate shorter versions of some popular measures in an older adult sample. The other issue within the literature is the use of relatively small sample sizes to investigate components of navigation ability. The majority of studies have sample sizes less than 100. For example, in a meta-analysis conducted by Techentin et al. (2014), 80 studies were identified, and only 3 of those had an older adult sample of 100 or more; the majority of studies had sample sizes of around 50 or less. Hence, in terms of identifying components of navigation, many studies lack power. Further, use of extensive test batteries runs the risk of fatiguing older adult participants, thereby depressing their performance, and reducing the likelihood of having adequate range on tests to identify predictors.

The current work is intended to address the major concerns described above, implementing a succinct assessment of navigation ability with a sizable sample of older adults. This study is a part of a multi-site center called ENHANCE (Enhancing Neurocognitive Health, Abilities, Networks, and Community Engagement; NIDILRR #90REGE0012-01-00) which focuses on older adults with MCI, traumatic brain injury (TBI), and post-stroke cognitive impairment (PSCI). The project the current data were collected for is called AUGMENT (Augmenting User Geocoordinates and Mobility with Enhanced Tutorials) and plans to develop instructional packages to assist impaired older adults in using popular navigation software. This project has three phases, with the first being a needs assessment. As a part of phase one, a protocol was developed to assess the issues cognitively impaired older adults experience with navigation and navigation aids during daily life. Before administering our protocol to the target populations, the protocol was administered to a community-dwelling sample of older adults in the southeastern United States. This was done both to confirm the viability of our protocol design and to validate novel measures of navigation ability. In doing so, we collected one of the largest single samples investigating navigation ability in an older adult population. The protocol consisted of administering two objective measures of navigation components, three shortened and novel self-report measures of navigation, and two self-report measures of memory ability.

The goal of the current analysis was to assess validity and reliability for a reduced set of navigation ability questions while exploring the conceptual structures of navigation with our novel measures. A second goal was to assess likely predictors of navigation ability, based on prior literature. To do so, measures of reliability were calculated for each shortened measure. The structure of the three self-report scales of navigation was assessed through the use of exploratory factor analysis (EFA). Lastly, three multiple regressions were conducted predicting each of the three self-report scales of navigation using a set of variables expected to be significant predictors based on prior literature.

## 2 Methods

### 2.1 Participants

Community-dwelling older adults were recruited from the Institute for Successful Longevity’s (ISL) online registry, a database containing contact information for approximately 2,600 older adults in the Leon, Gadsden, and Wakulla counties of northern Florida who had volunteered to participate in aging research. To be eligible for participation in this online study, individuals needed to be 60 years of age or older, be fluent in English, and have access to the internet. The latter requirement was necessary as the study was conducted during the COVID-19 pandemic when in-person testing was not permitted. In total, 450 individuals responded and completed the survey.

### 2.2 Materials

#### 2.2.1 Demographic questionnaires

A set of general questions not directly measuring navigation were administered. These included age, gender, education, race and ethnicity, and income (see Table 1).

**TABLE 1:** Participant characteristics.

Variables	*N*	%	*M*	*SD*	Range
Demographics					
Age			69.74	5.42	60–90
Gender					
Male	154	40.53	—	—	—
Female	226	59.47	—	—	—
Education					
High school graduate/GED	10	2.63	—	—	—
Vocational training	11	2.89	—	—	—
Some college/Associates degree	78	20.52	—	—	—
Bachelor’s degree (BA, BS)	88	23.16	—	—	—
Master’s (or other post-graduate training)	141	37.10	—	—	—
Doctoral degree (PhD, MD, EdD, DDS, JD, etc.)	50	13.16	—	—	—
Race/Ethnicity					
White/Caucasian	352	92.63	—	—	—
Black/African American	7	1.84	—	—	—
Asian	2	0.53	—	—	—
Multi-racial	2	0.53	—	—	—
Other	7	1.84	—	—	—
Hispanic					
Yes	5	1.31	—	—	—
No	373	97.11	—	—	—
Annual household income					
Less than $10,000	1	0.26	—	—	—
$10,000–$19,999	9	2.37	—	—	—
$20,000–$39,999	31	8.16	—	—	—
$40,000–$59,999	53	13.95	—	—	—
$60,000–$79,999	70	18.42	—	—	—
$80,000 or more	171	45.00	—	—	—
Objective assessments					
Spatial orientation test					
Average angle difference from correct response			60.37	32.76	8.93–129.29
Directions and orienting assessment			94.40%	10.24%	33%–100%
NSEW subscale			95.94%	11.92%	0%–100%
LR subscale			92.66%	14.24%	0%–100%
Self-report assessments					
Wayfinding ability			38.46	6.96	11–49
Memory ability			32.70	5.09	14–42
Severity of memory difficulties			11.59	6.12	6–37

#### 2.2.2 Self-reported memory performance

Participants were asked to report their self-perceived memory ability and the severity of any memory issues they currently experience. Items for these two scales were sampled from the “Metamemory Questionnaire” (Gilewski20141990). For self-reported memory performance, participants were given six statements and were asked to rate how applicable they were to themselves on a 7-point Likert scale where a 1 represented “Not at all” and a 7 represented “Fully.” Scores could range from 6 to 42, where a higher score meant better perceived memory ability. For the severity of memory issues currently experienced, participants were given an additional six statements to rate in a similar fashion to memory performance, again on a 7-point Likert scale. Here, a 1 represented “Not serious” and a 7 represented “Very serious.” Scores could range from 6 to 42, where a higher score indicated more severe issues with memory.

#### 2.2.3 Spatial orientation test

Also referred to as the Perspective Taking Task, the Spatial Orientation Test (SOT; Hegarty and Waller, 2004) is an objective measure of spatial ability in which participants must orient themselves in a two-dimensional space. Participants are given an image with a set of objects in the environment (a cat, a car, a flower, etc.). Questions instruct the participant that they are standing at the location of one object, facing a second object, and are tasked with drawing the angle at which a third object is located relative to their imagined position. The SOT consists of twelve questions. For the current project, a digital version of the SOT was used (Friedman et al., 2020).

#### 2.2.4 The directions and orienting assessment

As a part of this project, a novel objective measure of spatial orientation was developed. This first iteration, referred to as the DORA, asks participants to follow a list of directions to a specified intersection in a hypothetical neighborhood. At the final destination, participants are given a multiple-choice response to decide which of the given buildings is the closest to their location. The DORA consists of two subsections, based on the instructional variations. The first subsection lists the directions using cardinal directions (e.g., go north on 5th Street, east on 7th Avenue, etc.) while the second subsection lists the directions using left and right (e.g., turn left on 5th Street, turn right on 7th Avenue, etc.). Each subsection contains five questions for a total of ten questions.

#### 2.2.5 Navigation ability

To measure self-perceived ability to navigate, participants completed three novel subscales. Questions for these subscales were sampled from the “Wayfinding Questionnaire” (van der Ham et al., 2013; de Rooij et al., 2019) and the “Santa Barbara Sense of Direction Questionnaire” (Hegarty et al., 2002). Sampled items from these two scales were chosen based on relevancy to the target population and to the type of activity in question, that being older adults navigating from one location to another. For the first subscale, participants were asked how often they felt lost when moving around various environments. This subscale contained six items using similar wording to items 12-14 of the “Wayfinding Questionnaire,” and the size of the environment increased from item to item. Environments began as “Your yard, parking lot, or area surrounding your home” and ended at “Your region.” For each item, participants responded using a 5-point Likert scale where a 1 represented “Never” and a 5 represented “Always.” A higher score on the “Feeling Lost” subscale indicated greater issues with navigation.

For the second subscale, participants were asked how often they needed help from someone else when navigating various environments. The environments used for this subscale are identical to the “Feeling Lost” subscale, as is the scoring method and the chosen wording. Consequently, a higher score on the “Needing Help” subscale indicated a general greater desire for assistance when navigating.

The final subscale was referred to as the Wayfinding subscale. This reduced set consisted of seven items derived from both the “Wayfinding Questionnaire” and the Santa Barbara Sense of Direction Questionnaire, each one asking how applicable a given statement was to the participant. Each item was scored on a 7-point Likert scale where a 1 represented “Not at all” and a 7 represented “Fully.” Scores could range from 7 to 49, and a higher score indicated more confidence in their wayfinding ability.

### 2.3 Procedure

Participants were recruited from the ISL online registry. All participants were sent an email with an anonymous link to a Qualtrics survey containing the entire protocol. Participants completed the Qualtrics survey on their own without a given time limit. After giving electronic informed consent, participants first completed the demographic questionnaire section and the self-assessment questionnaires. After, participants completed the SOT and then the DORA. Once the entire survey was completed, participants were thanked for their time and given the option to enter a raffle for a $50 gift card.

### 2.4 Statistical analyses

All analyses were conducted using the R statistical package (Windows Version 4.1.2; R Core Team, 2013) with the *psych* (Revelle, 2017), *EFAtools* (Steiner and Grieder, 2020), and *factoextra* (Kassambara and Mundt, 2017).

To confirm the items pertaining to navigation were factorable, Bartlett’s test of sphericity (Bartlett, 1954) and the KMO statistic (Kaiser, 1974) were collected. Bartlett’s test was significant [χ^2^(171) = 4863.19, *p* < 0.001] and the overall KMO statistic was 0.874, indicating that the data to be factored were statistically sound to do so. Factor analysis was chosen because of the desire to investigate latent factors that may exist within the data (Fabrigar et al., 1999). The minimum residual factor method was used for extraction. This method was selected because it works best in situations where the true number of factors is unknown while providing equitable fits when compared to similar methods (Kline, 2014). As a check on the factorability of the given set of items, eigenvalues were calculated to determine the optimal number of factors. Following a recognized criterion (# of eigenvalues > 1), five factors were identified. This decision was further supported by the observation of the scree plot levelling off after five factors. Further, parallel analysis was conducted as an empirical check and suggested between 3 and 7 factors. EFA was tested at the 3-, 5-, and 7-factor level, and the 5-factor model accounted for the most variance while avoiding overfitting the data. Thus, we only report the findings from the 5-factor model. For determining adequacy of items for a given factor, given the sample size analyzed, a cutoff of 0.3 was used (Hair, 2009).

The higher-order factor analysis was conducted *post hoc* both due to the unexpectedness of the factoring as well as to determine if each lower-order factor still fit under the umbrella of navigation. To verify that the number of higher-order factors was one, the same process of generating eigenvalues and conducting a parallel analysis was used. This process suggested a single higher-order factor. To maintain consistency and for reasons stated earlier, the higher-order factor analysis utilized the same extraction and transformation methods.

For the multiple regression analyses, predictors were chosen based primarily on theoretical backing. These predictors would have the greatest likelihood of resulting in significant outcomes while conserving statistical power. Thus, three separate multiple regression analyses were conducted to assess if the independent variables of gender, age, SOT performance, DORA performance, self-reported memory difficulties, and self-reported severity of memory difficulties predicted each of the three navigation scales (i.e., “Needing Help,” “Feeling Lost,” and Wayfinding). Gender and age represented demographic information, SOT and DORA represented objective navigation measures, and memory represented a key cognitive ability for older adults.

## 3 Results

### 3.1 Descriptive statistics

After the data were collected, it was observed that some participants had taken an exceedingly long time to complete the online survey, some well over 4 h. To account for these data systematically, time limits were developed. To do so, the time taken to complete the SOT, time to complete the DORA, and time to complete the full survey were gathered. Interquartile ranges (IQR) were calculated, and 1.5 times above the IQR (1.5*IQR) was generated. From there, appropriate cutoffs were selected. For the SOT, the standard time limit is 5 min (Friedman et al., 2020). Given the SOT was developed for use with a younger population, the time limit was doubled to 10 min for the current sample, given that cognitive and motor processing cycle time is about twice as long for older compared to younger adults (Jastrzembski and Charness, 2007). For the novel DORA, 1.5*IQR was 17.05 min. That was then rounded to 20 min allotted for the DORA. Overall completion time generated a 1.5*IQR of 53.18 min which was rounded to 60 min. Thus, participants were allowed to take 10 min to complete the SOT, 20 min to complete the DORA, and 60 min to complete the entire survey.

Through this method of exclusion, 40 (8.9%) participants were excluded from the dataset^1^. 7 (1.6%) participants took too long on the SOT, 12 (2.7%) participants took too long on the DORA, and 32 (7.1%) participants took too long on the full survey. For the purpose of regression analyses, an additional 26 participants were excluded due to missing or incomplete data. Hence, the final participant total was 380. Table 1 provides descriptive statistics for the final sample.

### 3.2 Exploratory multi-level factor analysis

**FIGURE 1:**
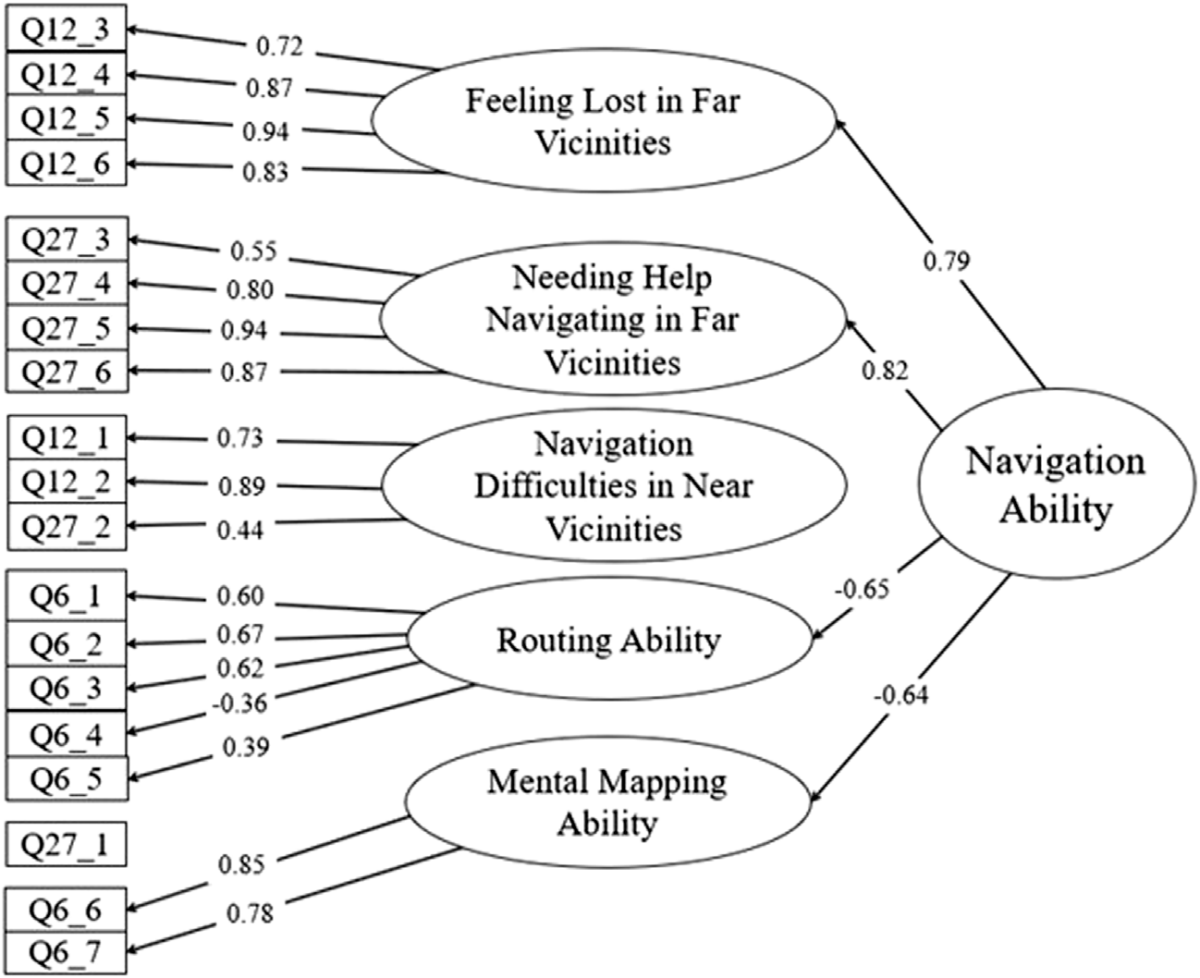
Multi-level factor analysis of navigation items. Questionnaire items are represented by boxes on the left while latent factors are displayed to the right as ovals. Items marked as “Q12” are from the “Feeling Lost” subscale. Items marked as “Q27” are from the “Needing Help” subscale. Items marked as “Q6” are from the Wayfinding subscale. Only loadings above 0.3 are displayed.

To obtain a better understanding of the construct validity and to observe any latent factors within the navigation subscales, an EFA was conducted. Due to the exploratory nature of the EFA, as well as the theoretical reasons, no items were dropped from the solution regardless of loadings. The minimum residual factor analysis contained 19 items, using oblimin transformations. With five factors, the EFA accounted for 61% of the variance. Figure 1 lists the loadings of the EFA. For clarity, a loading cutoff of 0.3 was used.

Of the 19 items, 11 of them had primary loadings over 0.7, indicating strong loadings on their respective factors. Four items had primary loadings between 0.5 and 0.7, and three items had primary loadings between 0.3 and 0.5. No cross-loadings exceeded 0.2. Only one item did not have a loading above 0.3, and that item was from the “Needing Help” subscale. Specifically, it asked “How often do you need help from someone else to find your way around: - Your yard, parking lot, or area surrounding your home.” This can likely be attributed to a strong floor effect; over 95% of responses indicated “Never” having issues in this category.

Upon observation of the generated factors, novel factor labels were created to best encapsulate the items loading on to said factor. Two distinct factors represented issues navigating in farther vicinities (e.g., your town, your county, your state, and your region) while a single composite factor represented issues navigating in near vicinities (e.g., the area around your home and your neighborhood). The wayfinding subscale was broken into two separate factors, one appearing to load items related to routing ability (e.g., one’s ability to generate paths to desired locations) and the other related to mental mapping (e.g., one’s self-perceived ability to orient in a three-dimensional space).

Because the three subscales were focused on navigation and appeared to factor in a unique way, a multi-order factor analysis was conducted to confirm these five factors loaded onto a single factor, named navigation. Thus, the previous EFA was replicated to include the multi-level factoring as a second step. The minimum residual multi-level factor analysis was conducted using oblimin transformations. The multi-level analysis accounted for 44% of the variance. As Figure 1 shows, 4 of the lower-level factors had loadings greater than 0.6. A single factor had a loading below the cutoff of 0.3, “Navigation Difficulties in Near Vicinities.” Following the logic used to name the lower-level factors, and because all the items being included were related to navigation, the higher-level factor was labeled “Navigation Ability.”

### 3.3 Reliability estimates for measures

For the three outcome variables of self-assessed navigation ability, the Wayfinding subscale was highly reliable (7 items; *α* = 0.85), as was the “Feeling Lost” subscale (6 items; *α* = 0.84) and the “Needing Help” subscale (6 items; *α* = 0.83).

With the collected sample, the predictor variable SOT was found to be highly reliable (12 items; *α* = 0.87). Self-assessed memory ability was also highly reliable (6 items; *α* = 0.84) as well as self-assessed severity of memory difficulties (6 items; *α* = 0.84). The DORA was found to have poor reliability (10 items; *α* = 0.52). The cardinal-direction subscale of the DORA had similarly poor reliability (5 items; *α* = 0.50) as did the Left-Right subscale (5 items; *α* = 0.33).

### 3.4 Multiple regression analyses

With the chosen predictors of navigation, the first model predicting performance on the wayfinding subscale was significant [*F*(6,373) = 25.55, *p* < 0.01, *r* ^*2*^_*adj*_ = 0.28]. Table 2 shows the impact of the individual predictors within the overall model. Gender was a significant predictor (*t* = −3.53, *p* < 0.01) such that men reported higher wayfinding ability than women. SOT performance was also significant (*t* = −2.66, *p* < 0.01), representing higher performance on the SOT indicated better reports on the wayfinding subscale. Self-assessed memory ability was another significant predictor (*t* = 6.82, *p* < 0.01), showing that individuals that reported having better memory ability also reported better wayfinding ability. Last, severity of memory difficulties was a significant predictor within the model (*t* = −3.48, *p* < 0.01) and showed that individuals reported lower severity of memory difficulties also reported having better wayfinding.

**TABLE 2:** Multiple regression predicting wayfinding subscale.

Individual predictors (Z-standardized)	*B* (*SE*)	*β*
Gender	−2.31 (0.65)**	−0.163
Age	−0.02 (0.06)	−0.012
SOT average angle difference	−0.03 (0.01)**	−0.126
DORA percent correct overall	5.17 (3.16)	0.076
Self-assessed memory ability	0.48 (0.07)**	0.354
Self-assessed severity of memory difficulties	−0.20 (0.06)**	−0.178

Note: **p < 0.01.

The second overall model predicting performance on the frequency of feeling lost subscale was significant [*F*(6,368) = 20.61, *p* < 0.01, *r* ^*2*^_*adj*_ = 0.24] and is detailed in Table 3. Gender was a significant predictor (*t* = 4.31, *p* < 0.01) such that men reported lower frequencies of feeling lost than women. SOT performance was significant (*t* = 2.64, *p* < 0.01) such that higher SOT performance indicated lower frequencies of feeling lost. Self-assessed memory ability (*t* = −6.38, *p* < 0.01) and severity of memory difficulties (*t* = 3.20, *p* < 0.01) were also significant predictors in the model.

**TABLE 3:** Multiple regression predicting feeling lost subscale.

Individual predictors (Z-standardized)	*B* (*SE*)	*β*
Gender	1.26 (0.29)**	0.206
Age	0.01 (0.03)	0.027
SOT average angle difference	0.01 (0.00)**	0.130
DORA percent correct overall	2.06 (1.41)	0.070
Self-assessed memory ability	−0.20 (0.03)**	−0.341
Self-assessed severity of memory difficulties	0.08 (0.03)**	0.169

Note: **p < 0.01.

When predicting performance on the frequency of needing help subscale, the overall model was significant [*F*(6,363) = 15.91, *p* < 0.01, *r* ^*2*^_*adj*_ = 0.20] and is detailed in Table 4. Gender was a significant predictor (*t* = 2.59, *p* = 0.01) such that men reported lower frequencies of needing help navigating than women. SOT performance was significant (*t* = 2.34, *p* = 0.01) such that higher SOT performance indicated lower frequencies of needing help navigating. Self-reported memory ability was a significant predictor (*t* = −5.22, *p* < 0.01) as well as severity of memories difficulties (*t* = 3.44, *p* < 0.01); this indicated that individuals with better self-assessments of memory performance also reported lower frequencies of needing help navigating.

**TABLE 4:** Multiple regression predicting needing help subscale.

Individual predictors (Z-standardized)	*B* (*SE*)	*β*
Gender	0.71 (0.27)*	0.128
Age	0.02 (0.02)	0.047
SOT average angle difference	0.01 (0.00)*	0.129
DORA percent correct overall	0.56 (1.33)	0.021
Self-assessed memory ability	−0.16 (0.03)**	−0.291
Self-assessed severity of memory difficulties	0.08 (0.02)**	0.189

Note: **p < 0.01; *p < 0.05.

## 4 Discussion

In the current study, 450 community-dwelling older adults completed an online survey containing questions and assessments related to cognition and navigation ability. Responses from a subset were used to investigate the validity and reliability of novel, shortened self-report measures of navigation. Those self-report measures of navigation were also predicted by several key factors known in the literature. To our knowledge, this is one of the largest single samples looking at community-dwelling older adults and navigation in the literature to date. While samples of this size present a challenge regardless of the protocols being conducted, we recommend online implementations of objective measures as well as subjective surveying if in-person testing is unavailable. Online administration presents a useful opportunity for fast, efficient data collection. However, such data collection methods come at the cost of reduced generalizability as technology proficiency in older adults is lower than that in younger adults (Faverio, 2022), so samples are likely to be biased, with fewer older adults with lower socio-economic status participating. Tools for measuring the proficiency of older adults at various technologies have been developed (Boot et al., 2015; Roque and Boot, 2018; Roque and Boot, 2021), and it is recommended that future research endeavors seek to account for this when appropriate.

### 4.1 Exploratory multi-level factor analysis

When examining the factor analyses, the results provided are unique. While one would initially expect three factors to have been found, in accordance with the three subscales used, the EFA produced five individual factors with no cross-loading. These five factors did not perfectly correspond with the given subscales. From the wayfinding subscale, two factors emerged; one factor included questions related to generating mental maps of one’s surrounding area while the other included questions on one’s ability to execute said routes. This differentiation seems to suggest these two behaviors as separate skills an individual implements when navigating to a destination.

Perhaps the more interesting finding from the EFA was the loadings produced from the other two subscales, “Feeling Lost” and “Needing Help” navigating. Three factors were generated from these two subscales, and the loadings appeared to differentiate based on the navigation distance. Questions from both subscales referring to nearer distances, including the area surrounding one’s home and one’s neighborhood, loaded onto a single factor while farther distances, ranging from within one’s city to one’s general region, loaded on another. As these findings are exploratory, replication is needed. They also suggest a generalization of difficulties when referring to nearer distances. This is represented by how two of the subscales, “Needing Help” and “Feeling Lost,” items that referred to nearer distances (e.g., in the immediate neighborhood) loaded onto a single factor. By contrast, items from these two subscales that referred to far distances (e.g., your county, state, or region) loaded onto two separate factors. These findings support the idea that navigation across farther distances and nearer distances are distinct skills. The data further support this conclusion due to the results of the higher-level factor analysis, where “Navigation Difficulties in Near Vicinities” was the only factor to not load onto navigation (see Figure 1). Nearer distances may be classifiable as a separate skill from navigating across farther distances. Nearer distances, at least the ones probed in our subscales, represent distances that are traversed on a regular basis; hence it would be sensible to consider navigating nearer distances as a form of automatized task. Far distances, on the other hand, are much less frequently traversed, so navigation in this context likely requires conscious planning; one must determine the end destination, generate a route, and execute said route with full attention being given to the task.

### 4.2 Reliability estimates

Because of the novel measures being used and to confirm that established measures were providing consistent data, reliability metrics were collected. Across most measures, reliability was found to be high. The exception was the novel DORA task, where reliability was poor both for the overall task and the subscales (cardinal and left-right directions). These poor reliability metrics are likely due to significant ceiling effects. Almost two-thirds of participants received a perfect score on the overall task. 86.3% of participants received perfect scores on the cardinal-direction subscale while 74.5% of participants received perfect scores on the left-right subscale. It will be necessary to set a time limit for DORA, based on the current completion time data, to avoid incurring ceiling effects in future samples.

### 4.3 Multiple regression analyses

The multiple regressions conducted here support previous findings in the literature. Across all regression analyses, gender, SOT performance, self-assessed memory ability, and self-assessed severity of memory difficulties were significant predictors. Age and DORA performance were not significant predictors in any of the three regressions. All regression analyses explained 20% or more of variance in the data of their respective subscale. While age not being significant isn’t a surprise, given those recruited were required to be 60 years of age or above, this finding supports no differentiation in navigation ability between young-old, middle-old, and oldest-old adults, a result that the literature has reported in other areas of cognition (Rhodes, 2004; Kliegel et al., 2008). While the DORA was not a significant predictor in any regression model, this is likely due to the low reliability metrics for both the overall task as well as the two subscales. In general, gender, SOT performance, and self-assessed severity of memory difficulties were similar in terms of strength of prediction (beta weights) for each regression. However, self-assessed memory ability was the strongest predictor in all three regression analyses.

### 4.4 Study limitations

While large, the analyzed sample was not representative of the general older adult population. It was predominantly white, well-off financially, and highly educated. This sampling is partially due to the demographics of the ISL registry, but it may also be due to the need for a degree of technology proficiency to access and complete the online survey. Future studies should seek to sample from a more diverse pool of participants in terms of a wider range of socioeconomic levels and ethnicities. We would also like to reiterate the exploratory nature of many of these analyses. While results show many of our reduced item sets were highly reliable, the EFA generated showed a more complex relationship between our item sets and general navigation ability.

It is also important to mention that much of the data is based on self-assessed or self-reported questionnaires. Namely, the measures of navigation and memory were self-reports. There is some discussion in the literature about exaggeration of memory performance and related cognition when self-assessed (Smith et al., 1996; Jungwirth et al., 2004; Willard and Gramzow, 2008). For future studies, it would be beneficial to collect objective and subjective measures for cognitive abilities to allow for better detection of potential correlations.

A major limitation for this study is the travel frequency and experience of the collected sample. As the data were collected during the height of the COVID-19 pandemic, travel for older adults was significantly less safe and restricted. While items in the navigation scales were phrased in such a way as to target general concepts (ex., “how often do you need help from someone else to find your way around?”), it is possible that the COVID-19 pandemic affected participant’s recent experiences with navigation. This is particularly likely when interpreting results related to traveling far distances. Additionally, this study does not report on how older adults choose to navigate their environment. Use of GPS and other assistive technologies may influence how individuals may self-report navigation difficulties, though the potential effect is not determinable with this sample. Past research has generally found negative effects of GPS reliance but the relationship is complicated (Steele, 2016; Ruginski et al., 2019). Future studies should aim to collect information on the methods older adults choose to use when navigating their environment and incorporate these strategy-level behaviors into analyses.

### 4.5 General discussion

In summary, the current work had two goals: to quantify the reliability and validity for novel measures of navigation and to probe likely predictors of self-assessed navigation. When assessing reliability and validity, we found robust support for the use of reduced question sets for navigation. All navigation subscales, with the exception of the DORA, containing only a handful of items each, were found to be highly reliable and, even with a reduced number of items, were able to generate a single higher-order factor (navigation ability) as well as multiple sub-factors using EFA. These findings help support the view that a diverse set of skills (such as spatial orientation, memory) are required to successfully navigate in one’s environment. In terms of validity assessment, regression analyses showed that the same set of measures were related to multiple facets of navigation ability and that previously identified correlates of navigation ability, for instance, gender, were also predictors here. Chief among those was self-assessed memory ability, indicating that memory processes are a crucial component in successful navigation, consistent with prior findings that hippocampal networks are involved in both memory and navigation ability.

Navigation and mobility are important facets of everyday living for everyone. These become even more so as we age. Maintaining mobility is connected with a range of outcomes, from cognitive function to general wellbeing (Rantakokko et al., 2013; De Silva et al., 2019). For this reason, it is paramount that research efforts continue to expand the literature in understanding the complexities of navigation. The present study demonstrates that multiple facets of a larger construct can be reliably measured with a handful of items and that navigation is a complex skill loading into different factors for consideration. For future studies, we suggest researchers implement reduced item sets, enabling more efficient data collection including probing more aspects of the navigation construct. Findings from the EFA suggest a complex relationship between potential subskills of navigation, and further investigation should aim to probe deeper into the individual skills that contribute to general navigation success in older adults. Finally, it would be useful to assess how well self-reports of navigation ability correlate with objectively assessed navigation performance.

## Data Availability

The raw data supporting the conclusion of this article will be made available by the authors, without undue reservation.

## Footnotes

1 Sensitivity analyses were conducted to check whether removal of these participants would affect outcome metrics. Including available data for all available participants did not alter the overall findings. Sensitivity analyses for the regressions have been included in Supplementary Material.

## References

[B1] Bartlett M. S. (1954). A note on the multiplying factors for various χ^2^ approximations. J. R. Stat. Soc. 16 (2), 296–298. 10.1111/j.2517-6161.1954.tb00174.x

[B2] Boot W. R. Charness N. Czaja S. J. Sharit J. Rogers W. A. Fisk A. D. (2015). Computer proficiency questionnaire: assessing low and high computer proficient seniors. Gerontologist 55 (3), 404–411. 10.1093/geront/gnt117 24107443 PMC4542703

[B3] Costa R. Q. M. D. Pompeu J. E. Viveiro L. A. P. D. Brucki S. M. D. (2020). Spatial orientation tasks show moderate to high accuracy for the diagnosis of mild cognitive impairment: a systematic literature review. Arq. Neuro-Psiquiatria 78, 713–723. 10.1590/0004-282X20200043 33331465

[B4] Coughlan G. Laczó J. Hort J. Minihane A. M. Hornberger M. (2018). Spatial navigation deficits—overlooked cognitive marker for preclinical Alzheimer disease? Nat. Rev. Neurol. 14 (8), 496–506. 10.1038/s41582-018-0031-x 29980763

[B5] De Rooij N. K. Claessen M. H. G. van der Ham I. J. Post M. W. M. Visser-Meily J. M. A. (2019). The Wayfinding Questionnaire: a clinically useful self-report instrument to identify navigation complaints in stroke patients. Neuropsychol. Rehabil. 29 (7), 1042–1061. 10.1080/09602011.2017.1347098 28720024

[B6] De Silva N. A. Gregory M. A. Venkateshan S. S. Verschoor C. P. Kuspinar A. (2019). Examining the association between life-space mobility and cognitive function in older adults: a systematic review. J. aging Res. 2019, 3923574. 10.1155/2019/3923574 31275650 PMC6589294

[B7] Fabrigar L. R. Wegener D. T. MacCallum R. C. Strahan E. J. (1999). Evaluating the use of exploratory factor analysis in psychological research. Psychol. methods 4 (3), 272–299. 10.1037/1082-989x.4.3.272

[B8] Faverio M. (2022). Share of those 65 and older who are tech users has grown in the past decade. Pew Research Center. Available at: https://www.pewresearch.org/fact-tank/2022/01/13/share-of-those-65-and-older-who-are-tech-users-has-grown-in-the-past-decade/ (Accessed July 21, 2022).

[B9] Friedman A. Kohler B. Gunalp P. Boone A. P. Hegarty M. (2020). A computerized spatial orientation test. Behav. Res. methods 52 (2), 799–812. 10.3758/s13428-019-01277-3 31347037

[B10] Gilewski M. J. Zelinski E. M. Schaie K. W. (1990). The Memory Functioning Questionnaire for assessment of memory complaints in adulthood and old age. Psychol. aging 5 (4), 482–490. 10.1037//0882-7974.5.4.482 2278670

[B11] Hair J. F. (2009). Multivariate data analysis. Kennesaw: Digital Commons Kennesaw State University.

[B12] Harada C. N. Love M. C. N. Triebel K. L. (2013). Normal cognitive aging. Clin. geriatric Med. 29 (4), 737–752. 10.1016/j.cger.2013.07.002 PMC4015335 24094294

[B13] Hegarty M. Richardson A. E. Montello D. R. Lovelace K. Subbiah I. (2002). Development of a self-report measure of environmental spatial ability. Intelligence 30 (5), 425–447. 10.1016/s0160-2896(02)00116-2

[B14] Hegarty M. Waller D. (2004). A dissociation between mental rotation and perspective-taking spatial abilities. Intelligence 32 (2), 175–191. 10.1016/j.intell.2003.12.001

[B15] Hort J. Laczó J. Vyhnálek M. Bojar M. Bureš J. Vlček K. (2007). Spatial navigation deficit in amnestic mild cognitive impairment. Proc. Natl. Acad. Sci. 104 (10), 4042–4047. 10.1073/pnas.0611314104 17360474 PMC1820705

[B16] Jastrzembski T. S. Charness N. (2007). The Model Human Processor and the older adult: parameter estimation and validation within a mobile phone task. J. Exp. Psychol. Appl. 13, 224–248. 10.1037/1076-898X.13.4.224 18194048 PMC4591021

[B17] Jungwirth S. Fischer P. Weissgram S. Kirchmeyr W. Bauer P. Tragl K. H. (2004). Subjective memory complaints and objective memory impairment in the Vienna‐Transdanube aging community. J. Am. Geriatrics Soc. 52 (2), 263–268. 10.1111/j.1532-5415.2004.52066.x 14728638

[B18] Kaiser H. F. (1974). An index of factorial simplicity. psychometrika 39 (1), 31–36. 10.1007/bf02291575

[B19] Kassambara A. Mundt F. (2017). Package factoextra. Extr. Vis. results Multivar. data analyses 76 (2).

[B20] Kliegel M. Jäger T. Phillips L. H. (2008). Adult age differences in event-based prospective memory: a meta-analysis on the role of focal versus nonfocal cues. Psychol. aging 23 (1), 203–208. 10.1037/0882-7974.23.1.203 18361667

[B21] Kline P. (2014). An easy guide to factor analysis. Oxfordshire: Routledge.

[B22] Lester A. W. Moffat S. D. Wiener J. M. Barnes C. A. Wolbers T. (2017). The aging navigational system. Neuron 95 (5), 1019–1035. 10.1016/j.neuron.2017.06.037 28858613 PMC5659315

[B23] Lithfous S. Dufour A. Després O. (2013). Spatial navigation in normal aging and the prodromal stage of Alzheimer's disease: insights from imaging and behavioral studies. Ageing Res. Rev. 12 (1), 201–213. 10.1016/j.arr.2012.04.007 22771718

[B24] Moffat S. D. (2009). Aging and spatial navigation: what do we know and where do we go? Neuropsychol. Rev. 19 (4), 478–489. 10.1007/s11065-009-9120-3 19936933

[B25] Plácido J. de Almeida C. A. B. Ferreira J. V. de Oliveira Silva F. Monteiro-Junior R. S. Tangen G. G. (2022). Spatial navigation in older adults with mild cognitive impairment and dementia: a systematic review and meta-analysis. Exp. Gerontol. 165, 111852. 10.1016/j.exger.2022.111852 35644416

[B26] R Core Team (2013). R: a language and environment for statistical computing. R Core Team.

[B27] Rantakokko M. Portegijs E. Viljanen A. Iwarsson S. Rantanen T. (2013). Life-space mobility and quality of life in community-dwelling older people. J. Am. Geriatrics Soc. 61 (10), 1830–1832. 10.1111/jgs.12473 24117303

[B28] Revelle W. R. (2017). PSYCH: procedures for personality and psychological research.

[B29] Rhodes M. G. (2004). Age-related differences in performance on the Wisconsin card sorting test: a meta-analytic review. Psychol. aging 19 (3), 482–494. 10.1037/0882-7974.19.3.482 15382998

[B30] Roque N. A. Boot W. R. (2018). A new tool for assessing mobile device proficiency in older adults: the mobile device proficiency questionnaire. J. Appl. Gerontology 37 (2), 131–156. 10.1177/0733464816642582 PMC9394541 27255686

[B31] Roque N. A. Boot W. R. (2021). A new tool for assessing older adults’ wireless network proficiency: the wireless network proficiency questionnaire. J. Appl. Gerontology 40 (5), 541–546. 10.1177/0733464820935000 PMC8363155 32623926

[B32] Ruginski I. T. Creem-Regehr S. H. Stefanucci J. K. Cashdan E. (2019). GPS use negatively affects environmental learning through spatial transformation abilities. J. Environ. Psychol. 64, 12–20. 10.1016/j.jenvp.2019.05.001

[B33] Smith G. E. Petersen R. C. Ivnik R. J. Malec J. F. Tangalos E. G. (1996). Subjective memory complaints, psychological distress, and longitudinal change in objective memory performance. Psychol. aging 11 (2), 272–279. 10.1037//0882-7974.11.2.272 8795055

[B34] Steele S. J. (2016). Predicting GPS usage: the relative importance of wayfinding ability, object-based spatial ability, working memory capacity, anxiety, and overall technology usage. Alabama: The University of Alabama.

[B35] Steiner M. D. Grieder S. (2020). EFAtools: an R package with fast and flexible implementations of exploratory factor analysis tools. J. Open Source Softw. 5 (53), 2521. 10.21105/joss.02521

[B36] Techentin C. Voyer D. Voyer S. D. (2014). Spatial abilities and aging: a meta-analysis. Exp. aging Res. 40 (4), 395–425. 10.1080/0361073X.2014.926773 25054640

[B37] Van der Ham I. J. Kant N. Postma A. Visser-Meily J. M. (2013). Is navigation ability a problem in mild stroke patients? Insights from self-reported navigation measures. J. rehabilitation Med. 45 (5), 429–433. 10.2340/16501977-1139 23615778

[B38] Van der Ham I. J. M. Claessen M. H. G. Evers A. W. M. van der Kuil M. N. A. (2020). Large-scale assessment of human navigation ability across the lifespan. Sci. Rep. 10, 3299. 10.1038/s41598-020-60302-0 32094394 PMC7039892

[B39] VanSwearingen J. M. Studenski S. A. (2014). Aging, motor skill, and the energy cost of walking: implications for the prevention and treatment of mobility decline in older persons. Journals Gerontology Ser. A Biol. Sci. Med. Sci. 69 (11), 1429–1436. 10.1093/gerona/glu153 PMC4271095 25182600

[B40] Willard G. Gramzow R. H. (2008). Exaggeration in memory: systematic distortion of self-evaluative information under reduced accessibility. J. Exp. Soc. Psychol. 44 (2), 246–259. 10.1016/j.jesp.2007.04.012

