# Supplementary material for: Components of navigation ability and their predictors in a community-dwelling sample of older adults
Source: Front Aging. 2023 Oct 19;4:1239094. doi: 10.3389/fragi.2023.1239094 (PMC10620738; doi:10.3389/fragi.2023.1239094)
Supplement: Supplementary file 1 [file Table1.DOCX]

Each of the regression analyses reported in the main manuscript were replicated utilizing all 450 participants, when possible. Generally, results from all sensitivity analyses replicated fully. Each of the predictors were still significant when including all possible data for their respective predicant (e.g., Wayfinding, “Feeling Lost”, and “Needing Help”).

For the first model predicting outcomes on the wayfinding subscale, the overall model was significant (*F*(6,409) = 26.87, p < 0.01, *r^2^_adj_* = 0.27). For the second model predicting outcomes on the “Feeling Lost” subscale, the overall model was significant (*F*(6,409) = 20.57, p < 0.01, *r^2^_adj_* = 0.22). For the third model predicting outcomes on the “Needing Help” subscale, the overall model was significant (*F*(6,409) = 17.24, p < 0.01, *r^2^_adj_* = 0.19). For all three models, gender, SOT performance, subjective memory ability, and subjective severity of memory issues were significant predictors. Supplemental Tables 1 through 3 provide additional details for individual predictors.

# **Supplemental Table 1.** Multiple Regression Predicting Wayfinding Subscale

| Individual Predictors (Z-Standardized) | *B (SE)* | *β* |
| --- | --- | --- |
| Gender | -2.42 (0.62)** | -0.173 |
| Age | -0.00 (0.04) | -0.002 |
| SOT Average Angle Difference | -0.03 (0.01)** | -0.134 |
| DORA Percent Correct Overall | 4.99 (2.98) | 0.075 |
| Self-Assessed Memory Ability | 0.48 (0.07)** | 0.349 |
| Self-Assessed Severity of Memory Difficulties | -0.19 (0.06)** | -0.168 |

*Note:* ***p* < 0.01

# **Supplemental Table 2.** Multiple Regression Predicting Feeling Lost Subscale

| Individual Predictors (Z-Standardized) | *B (SE)* | *β* |
| --- | --- | --- |
| Gender | 1.16 (0.28)** | 0.192 |
| Age | 0.01 (0.02) | 0.031 |
| SOT Average Angle Difference | 0.01 (0.00)** | 0.130 |
| DORA Percent Correct Overall | 2.06 (1.33) | 0.071 |
| Self-Assessed Memory Ability | -0.21 (0.03)** | -0.350 |
| Self-Assessed Severity of Memory Difficulties | 0.07 (0.02)** | 0.139 |

*Note:* ***p* < 0.01

# **Supplemental Table 3.** Multiple Regression Predicting Needing Help Subscale

| Individual Predictors (Z-Standardized) | *B (SE)* | *Β* |
| --- | --- | --- |
| Gender | 0.67 (0.26)** | 0.122 |
| Age | 0.02 (0.02) | 0.054 |
| SOT Average Angle Difference | 0.01 (0.00)** | 0.133 |
| DORA Percent Correct Overall | 0.63 (1.25) | 0.024 |
| Self-Assessed Memory Ability | -0.15 (0.03)** | -0.284 |
| Self-Assessed Severity of Memory Difficulties | 0.09 (0.02)** | 0.195 |

*Note:* ***p* < 0.01
